# Variations in Values of State, Response Entropy and Haemodynamic Parameters Associated with Development of Different Epileptiform Patterns during Volatile Induction of General Anaesthesia with Two Different Anaesthetic Regimens Using Sevoflurane in Comparison with Intravenous Induct: A Comparative Study

**DOI:** 10.3390/brainsci10060366

**Published:** 2020-06-12

**Authors:** Michał Stasiowski, Anna Duława, Izabela Szumera, Radosław Marciniak, Ewa Niewiadomska, Wojciech Kaspera, Lech Krawczyk, Piotr Ładziński, Beniamin Oskar Grabarek, Przemysław Jałowiecki

**Affiliations:** 1 Department of Anaesthesiology and Intensive Therapy, Faculty of Medical Sciences in Zabrze, Medical University of Silesia, 40-055 Katowice, Poland; iza_sz@vp.pl (I.S.); radeqm@gmail.com (R.M.); lech.kraw@gmail.com (L.K.); olaf@pro.onet.pl (P.J.); 2 Department of Anaesthesiology and Intensive Care, Railway District Hospital Katowice, 40-055 Katowice, Poland; adulawa@gmail.com; 3 Department of Epidemiology and Biostatistics, School of Public Health in Bytom, Medical University of Silesia, 40-055 Katowice, Poland; e.j.niewiadomska@gmail.com; 4 Department of Neurosurgery, Regional Hospital in Sosnowiec, Faculty of Medical Sciences in Katowice, Medical University of Silesia, 40-055 Katowice, Poland; wkaspera@sum.edu.pl (W.K.); jladzinska@polsoft.pl (P.Ł.); 5 Department of Clinical Trials, Maria Sklodowska-Curie National Research Institute of Oncology Krakow Branch, 31-115 Katowice, Poland; bgrabarek7@gmail.com; 6 Department of Histology, Cytophysiology and Embryology, Faculty of Medicine in Zabrze, University of Technology in Katowice, 41-800 Zabrze, Poland

**Keywords:** epileptiform EEG patterns, sevoflurane, response entropy, state entropy, rhythmic polyspikes, polyspikes, periodic epileptiform discharges

## Abstract

Background and Objectives: Raw electroencephalographic (EEG) signals are rarely used to monitor the depth of volatile induction of general anaesthesia (VIGA) with sevoflurane, even though EEG-based indices may show aberrant values. We aimed to identify whether response (RE) and state entropy (SE) variations reliably reflect the actual depth of general anaesthesia in the presence of different types of epileptiform patterns (EPs) in EEGs during induction of general anaesthesia. Materials and Methods: A randomized, prospective clinical study was performed with 60 patients receiving VIGA using sevoflurane with the increasing concentrations (group VIMA) or the vital capacity (group VCRII) technique or an intravenous single dose of propofol (group PROP). Facial electromyography (fEMG), fraction of inspired sevoflurane (FiAA), fraction of expired sevoflurane (FeAA), minimal alveolar concentration (MAC) of sevoflurane, RE and SE, and standard electroencephalographic evaluations were performed in these patients. Results: In contrast to periodic epileptiform discharges, erroneous SE and RE values in the patients’ EEGs were associated with the presence of polyspikes (PS) and rhythmic polyspikes (PSR), which were more likely to indicate toxic depth rather than false emergence from anaesthesia with no changes in the FiAA, FeAA, and MAC of sevoflurane. Conclusion: Calculated RE and SE values may be misleading during VIGA when EPs are present in patients’ EEGs. During VIGA with sevoflurane, we recommend monitoring raw EEG data in scientific studies to correlate it with potentially erroneous RE and SE values and the end-tidal concentration of sevoflurane in everyday clinical practice, when monitoring raw EEG is not available, because they can mislead anaesthesiologists to reduce sevoflurane levels in the ventilation gas and result in unintentional true emergence from anaesthesia. Further studies are required to investigate the behaviour of EEG-based indices during rapid changes in sevoflurane concentrations at different stages of VIGA and the influence of polyspikes and rhythmic polyspikes on the transformation of EEG signals into a digital form.

## 1. Introduction

General anaesthesia is sometimes associated with unwelcome events. Both volatile induction of general anaesthesia (VIGA) with sevoflurane and intravenous induction of general anaesthesia with propofol may induce seizure-like movements or seizures accompanied by haemodynamic instability [1,2], which are defined as clinically manifest events with confirmatory electroencephalographic patterns. Seizures during VIGA with sevoflurane were observed with an incidence of 5% in children [2], whereas subclinical activity in children’s electroencephalographic recordings (without clinically manifest events) during VIGA with sevoflurane have been reported to appear in 20% of cases [1,3], as compared to 47% in adults breathing spontaneously [4]. The appearance of these findings during VIGA with sevoflurane in children is known to increase to 88% with controlled hyperventilation and even up to 100% with hypocapnia [2]. Epileptiform patterns (EPs) in patients’ electroencephalographic recordings (EEGs) tend to appear later in the course of the VIGA with 6% and with 8% sevoflurane in children [5] and in adults [6], respectively. Their appearance in children corresponded to a surgical minimal alveolar concentration (MAC) of 1.75 with a median sevoflurane concentration of 4.3% causing major epileptiform signs [7].

Intraoperative awareness during general anaesthesia is a distressing complication with the potential for long-term psychological consequences [8], and the incidence of explicit recall in the Western world has been reported to be between 0.1% and 0.2% in the general surgical population and up to 1%-2% in patients at high risk for this complication [9]. Therefore, monitoring the depth of general anaesthesia is slowly becoming a worldwide standard despite the associated extra cost of anaesthesia. Monitors evaluating the hypnotic component of anaesthesia by analysing electroencephalogram (EEG) data may help reduce the incidence of intraoperative awareness with recall [10,11,12,13,14]. Response entropy (RE), state entropy (SE) and bispectral index (BIS) are the most commonly utilised parameters to monitor the depth of general anaesthesia [15] and are exemplary quantitative EEG-based indices [16]. The Datex-Ohmeda S/5 Entropy Module (Datex-Ohmeda Division, Instrumentarium Corp., Helsinki, Finland) was the first commercial monitor based on the two entropy-related indices: the state entropy (SE; range 0–91) and the response entropy (RE; range 0–100) [17]. Response entropy (RE) is calculated from EEG and the frontal electromyogram (fEMG), while state entropy (SE) is calculated mainly from EEG [18].

Electroencephalographic (EEG) signals are derived from a frontal sensor, which is quickly and easily applied to the patient’s forehead and does not entail complex preoperational preparations. The EEG signal is then transformed to a digital score between 0 and 100 visible on the anaesthetic monitor to control the hypnotic component of general anaesthesia: values over 80 indicate an awake state; between 60 and 80, sedation; between 40 and 60, a level of unconsciousness suitable for surgery; between 30 and 40, too deep anaesthesia; and under 30, overdose of anaesthetic [19,20]. This analysis usually serves as an addition to raw (EEG) analysis. EEG is an instrumental method very commonly used to evaluate cerebral electrical activity. Spontaneous electrical activity of the brain is recorded from the scalp and is correlated with the underlying brain function [21]. However, pre-operational procedures to fix electrodes on patient’s scalp are time-consuming and interpretation of EEG patterns is challenging even for experienced anaesthesiologists. Raw EEG signals are being increasingly used to monitor the depth of VIGA with sevoflurane because EEG-based indices are reported to show aberrant values [4,14,22,23,24,25].

BIS values during the presence of EPs on the EEG [12] under GA with sevoflurane were reported to either increase [6] or decrease [26] along with the hemodynamic instability characteristic of clinically manifest seizures [27] during both induction [28] of and recovery [29,30,31,32,33] from general anaesthesia. Although observance based on variations in BIS values during GA seems to be a useful EEG measure of sevoflurane drug effect, the superiority of its use over an anaesthetic concentration-guided protocol is still controversial [34], because conflicting anaesthetic management is even suggested by simultaneous BIS readings obtained 10.7% of the times when two different BIS devices were placed concurrently on the same patient. [35]. Similarly, SE showed a clinically significant degree of disagreement when probes were applied on both sides of the forehead in the same patient, since 4% of the simultaneously measured pairs of SE indicated different anaesthetic depths and differed by more than 10 points [36]. SE has been proven to be more likely than BIS to falsely indicate consciousness between the loss of consciousness and unconsciousness stages [37], whereas response entropy (RE) was found to indicate emergence from anaesthesia earlier than SE or BIS [18]. 

The abovementioned results suggest that EEG-based indices do not always provide a reliable measure of anaesthetic depth; hence, anaesthesiologists should not rely exclusively on the BIS and SE readings when assessing depth of anaesthesia. We had previously conducted a comparative, prospective study to assess the influence of VIGA with sevoflurane using two different techniques and intravenous induction of general anaesthesia using propofol on the possible presence of EPs [25]. This study additionally reports on the previous study [25]. As an expansion of this abovementioned study that was carried out in our Department of Anaesthesiology and Intensive Therapy, the aim of the current analysis, being an additional reporting, was to determine whether RE and SE index variations reliably reflect the actual depth of general anaesthesia during the presence of different types of EPs in patients’ EEGs during VIGA with sevoflurane using two different techniques and intravenous induction of general anaesthesia with a single dose of propofol. 

## 2. Materials and Methods

Patients who were scheduled to undergo elective orthopaedic knee surgery under combined general and regional anaesthesia in the Clinic of Orthopaedics at St. Barbara’s Memorial Hospital no. 5 in Sosnowiec, Poland, and met the inclusion criteria were asked to participate in the study. Sixty adult patients under 70 years of age with American Society of Anaesthesiologists (ASA) scores of I-II were enrolled after obtaining written informed consent. Ethical approval for this study (NN-6501-196/06) was provided by the Ethical Committee of Medical University of Silesia on December 19, 2006; the study was registered in the Clinical Trial Registry (SilesianMUKOAIIT6, NCT03209323).

The exclusion criteria were as follows: a history of epilepsy, medical treatment that might interfere with the EEG assessments (e.g., tranquilizers or antiepileptic drugs), or a neurological disease or a neurosurgical operation that would impair EEG or entropy EEG monitoring; pregnancy; drug or alcohol abuse; a history of pulmonary disease; or signs predicting difficult mask ventilation or intubation. To exclude any pre-existing epileptic EEG patterns, standard 30 min initial EEG recordings were performed for all patients participating in the study. The initial EEG was taken in a dark quiet room for 5 min as a baseline, followed by three eye-opening and closing sequences of 10 s each and photostimulation lasting 10 min (flash stimuli at frequencies of 3/6/9/12 Hz–alpha; 15/18/21/24 Hz–beta). Then, another baseline reading was obtained, and the patients were asked to achieve a state of hyperventilation by taking 20 forceful breathes per minute for five minutes. Finally, another baseline reading was obtained. Only patients without EPs in their EEGs were included into final analysis (Prof. Ville Jäntti). The fifty-five patients included in the study were randomly assigned to one of the three groups (A, B, or C) using blind random allocation via sealed envelopes. 

### 2.1. Induction of Anaesthesia

Before the induction of anaesthesia, none of the patients received any medication on the day of surgery. Directly before surgery, the patients were preoxygenated for 5 min with 100% oxygen and intravenously administered 10 mL/kg per body weight of Ringer solution. The patients in the VIMA group were anaesthetised with sevoflurane using the increasing concentrations technique. The patient was breathing spontaneously via a face mask and the sevoflurane concentration in the inhaled gas was doubled every 10 breaths, starting from 0.3 vol% and proceeding in the sequence 0.3–0.6–1.2–2.4–4.8–8 vol.% until a minimal alveolar concentration (MAC) of 2 was obtained in the exhalation gas. The patients in the VCRII group were anaesthetised with 8% sevoflurane and 92% oxygen using the vital capacity technique. The anaesthetic circuit was prefilled with 8% sevoflurane, and a face mask was applied to the patient’s face to allow them to breathe spontaneously. In group C, the patients were preoxygenated with 100% oxygen and received propofol intravenously at a single dose of 2.5 mg/kg of body weight with subsequent propofol infusion at an infusion speed of 4 mg/kg body weight/hour. Assisted ventilation was initiated in all groups after loss of consciousness (LOC) when hypoventilation appeared and the ciliary reflex disappeared. When the minimum MAC of 2% was reached for sevoflurane in patients from the VIMA and VCRII groups or 5 min after the bolus of propofol with subsequent propofol infusion in patients in the PROP group, the patients in all groups were paralysed with a standard intravenous dose of 0.08–0.1 mg/kg cisatracurium (Nimbex, Glaxo), and a laryngeal mask (LMA) was applied after 45 s. CO_2_ was maintained at 35–45 mmHg since hyperventilation may trigger epileptiform activity before and during the induction of general anaesthesia. After LMA placement, before the operation started, sevoflurane concentration was maintained at the level of 1 MAC. 

Throughout anaesthesia induction and surgery, standard monitoring procedures were utilised and close attention was paid to vital parameters such as non-invasive arterial pressure (BP), heart rate (HR), standard electrocardiography (ECG) II, arterial blood saturation (SaO_2_), fraction of inspired oxygen in the gas mixture (FiO_2_), facial electromyography (fEMG), fraction of inspired sevoflurane (FiAA), fraction of expired sevoflurane (FeAA), exhaled carbon dioxide concentration (EtCO_2_), minimal alveolar concentration of sevoflurane (MAC), and depth of anaesthesia using response entropy (RE) and state entropy (SE) (the Datex-Ohmeda S/5 Entropy Module; Datex-Ohmeda Division, Instrumentarium Corp., Helsinki, Finland) [38].

### 2.2. Electroencephalography and Entropy Analysis

The data were collected and digitised at 10 s intervals using the S5/Collect (Datex-Ohmeda Division) and Bispectrum Analyzer for BIS (BSA for BIS version 3.22B2 for A-2000—S. Hagihira) installed on a notebook computer (Fujitsu-Siemens Amilo Pi1515). Four EEG channels were recorded using Ag/AgCl_2_ cup electrodes (Spes Medica) attached to the scalp with EC2 Electrode Cream (Grass Technologies). The electrodes (Aspect Medical Systems, Natick, MA, USA) were positioned on both temporal bones lateral to the eyes, on both mastoid bones, on Fp1, Fp2 (international 10–20 electrode system) and Fpz, and the ground electrode was positioned in the centre of the forehead between the eyebrows. Four-channel EEG was recorded using the following electrode pairs: Fp1—left mastoid, Fp2—right mastoid, Fpz—left temporal, and Fpz—right temporal [4] (in accordance with the suggestions provided by Prof. Ville Jäntti). The impedance was set below 1 kΩ, and the electrodes were attached to the S/5 E-EEG module of the anaesthetic monitor S/5 (GE Healthcare). The SE and RE scores were derived from a sensor (Aspect Medical Systems) positioned diagonally on the patients’ foreheads according to the manufacturer’s instructions. 

Data recording started 5 min before induction of general anaesthesia and continued throughout the induction until the patient was paralysed with an intravenous neuromuscular blocking drug (NMBD). Since NMBDs are reported to decrease the BIS value [39], the moment of administration of NMBD constituted the end of the induction of anaesthesia in all the experimental groups. The EEGs recorded before and during the general anaesthesia induction were analysed offline by a neurophysiologist with expertise in monitoring anaesthetic EEGs, the recording technique, and the anaesthetic agent used (Prof. Ville Jäntti). Neurophysiological analysis of EEGs may show different patterns; however, only polyspikes (PS), rhythmic polyspikes (PSR), and periodic epileptiform discharges (PED) were considered to be of the epileptiform type in accordance with the suggestions provided by Prof. Ville Jäntti [20,21].

### 2.3. Statistical Analyses

Statistical analyses were performed using MS Excel, STATISTICA 12, Stat Soft (Cracow, Poland), and R 3.3.2, GNU General Public License. The measured data were characterized using the mean and standard deviation (X ± SD) as well as medians with interquartile range (M IQR). Normality of distribution was checked with the Shapiro–Wilk W test. The significance of differences between means was tested using ANOVA for multiple groups, and for skewed distributions, their compatibility in groups was examined using the Kruskal–Wallis test by ranks. Comparison of average values of tested parameters at different stages was performed via one-way analysis of variance (ANOVA) for repeated measurements and the Friedman Rank Sum test. Two-way analysis of variance (ANOVA) for repeated measures and the nonparametric aligned rank test for repeated measures were conducted in two-factor analysis, with the group and the stage of the study serving as factors. For nominal data, we used percentages. Relationships between nominal variables were verified by the Fisher’s exact test for n by m tables. Evaluation of the relationship between two continuous variables was performed using the Spearman correlation coefficient R’ with an appropriate test of significance. Statistical significance was set at the level *p* < 0.05 (NS, not statistically significant).

## 3. Results

Out of the total of 60 patients allocated in the current study, 20 patients from the VIMA group, 20 from the VCRII group, and 20 from the PROP group were included in further analyses. Four patients each from the VIMA and VCRII groups were excluded due to possible artefacts in their EEG signals (possibly as a result of asymmetric cup electrode placement or muscle artefacts causing high fEMG values). Patients in the VIMA, VCRII, and PROP groups showed no significant differences in terms of age (42.9 ± 14.5, 39.1 ± 13.2, and 42.6 ± 15.0 years, respectively; *p* = 0.664), body weight (74.9 ± 15.5, 76.5 ± 16.9, and 75.3 ± 15.3 kg, respectively; *p* = 0.950), height (168.1 ± 10.6, 173.5 ± 10.8, and 169.9 ± 10.1 cm, respectively; *p* = 0.302), and female-to-male participant ratios (6:10, 4:15, and 6:14, respectively; *p* < 0.05). In the VCRII group, EPs were registered in 7 (21.9%) patients, while in the VIMA group, EPs were registered in 10 (31.3%) patients. 

The patients’ parameters were analysed during the following six stages of VIGA: stage 1, onset of VIGA; stage 2, after LOC defined as absence of ciliary reflex; stage 3, 10 s before appearance of the very first EP; stage 4, the first 10 s of the very first EP; stage 5, when the highest RE and SE values appeared in the presence of EPs; and stage 6, when the highest RE and SE values appeared at least 10 s after disappearance of EPs. The time intervals from the start of VIGA (stage 1) to the very first EP in the EEG (stage 4) were significantly different between the VIMA and VCRII groups (VIMA group: 626 ± 241 s vs. VCRII group: 209 ± 195 s; *p* = 0.0043). In the VCRII group, EPs were registered in 7 (21.9%) patients, while in the VIMA group, EPs were registered in 10 (31.3%) patients. 

The patients’ parameters were analysed during the following six stages of VIGA: stage 1, onset of VIGA; stage 2, after LOC defined as absence of ciliary reflex; stage 3, 10 s before appearance of the very first EP; stage 4, the first 10 s of the very first EP; stage 5, when the highest RE and SE values appeared in the presence of EPs; and stage 6, when the highest RE and SE values appeared at least 10 s after disappearance of EPs. The time intervals from the start of VIGA (stage 1) to the very first EP in the EEG (stage 4) were significantly different between the VIMA and VCRII groups (VIMA group: 626 ± 241 s vs. VCRII group: 209 ± 195 s; *p* = 0.0043).

**Figure 1:**
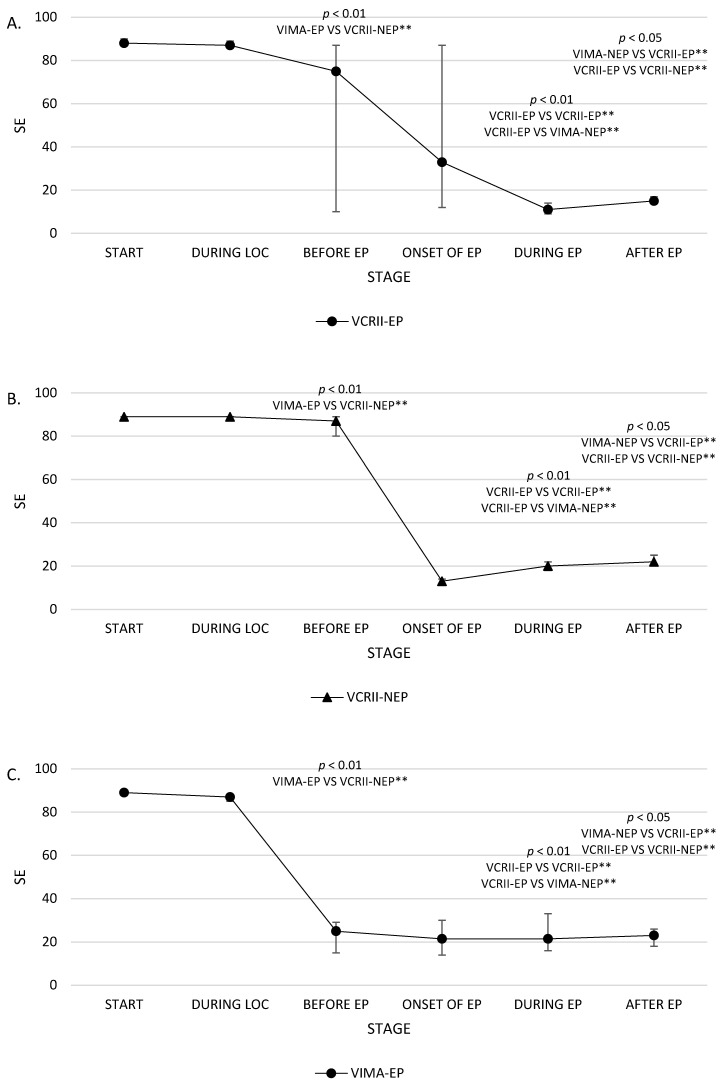

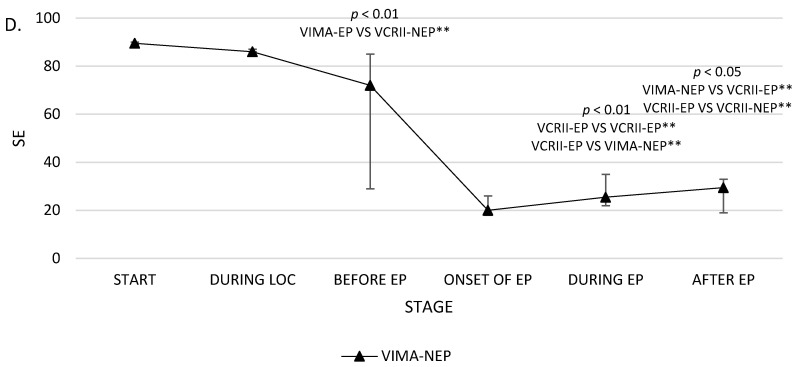
Medians with interquartile range (M (IQR)) of state entropy (SE) in groups: (**A**) volatile-vitalcap-EPI (VCRII-EP), (**B**) volatile-vitalcap-NOTEPI (VCRII-NEP), (**C**) (VIMA-EP), (**D**) (VIMA-NEP), on separate stages of the study, indicating a sequence of events (*p*-values: * <0.05, ** <0.01).

**Figure 2:**
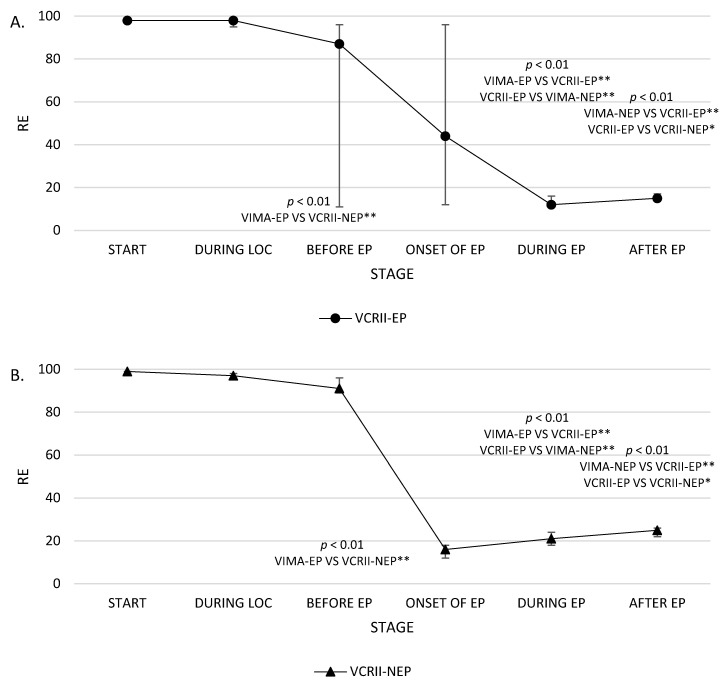

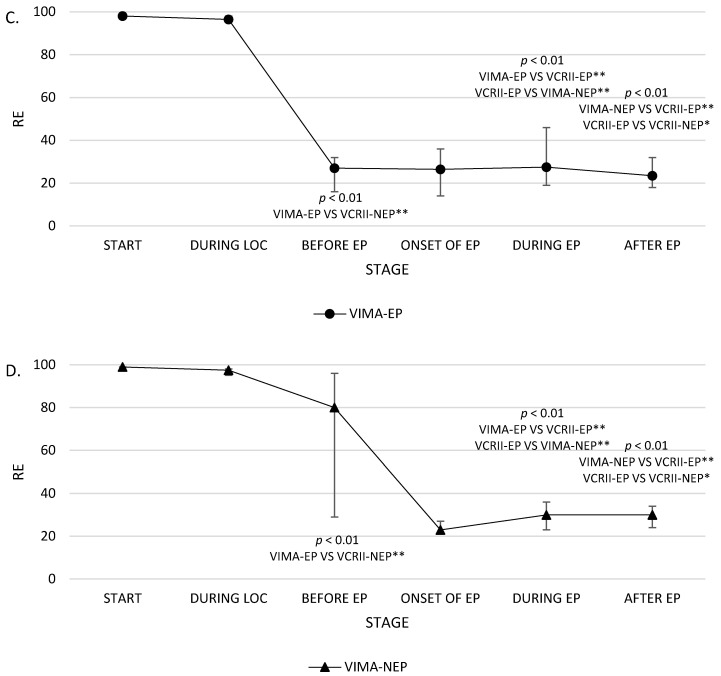
Median values M (IQR) of response entropy (RE) in groups: (**A**) volatile-vitalcap-EPI (VCRII-EP), (**B**) volatile-vitalcap-NOTEPI (VCRII-NEP), (**C**) (VIMA-EP), (**D**) (VIMA-NEP), on separate stages of the study, indicating a sequence of events (*p*-values: * <0.05, ** <0.01).

Additionally, the time range when EPs are bound to appear was calculated, and the vital parameters in both the groups were compared and analysed at six points in time: (1) before the induction of anaesthesia (stage 1); (2) after loss of consciousness (LOC) (stage 2); (3) before the presence of the first EP in the EEGs in the VIMA-EP and VCRII-EP groups, and the mean time to the potential presence of the first EP in the EEGs in the VIMA-nEP and VCRII-nEP groups (stage 3); (4) during the presence of the first EP in the EEGs in the VIMA-EP and VCRII-EP groups and during the mean time interval to the potential presence of the first EP in the EEGs in the VIMA-nEP and VCRII-nEP groups (stage 4); (5) during the presence of the highest RE and SE values associated with the first EP in the EEGs in the VIMA-EP and VCRII-EP groups and the highest RE and SE values in the EEGs in the VIMA-nEP and VCRII-nEP groups; and (6) during the presence of the highest RE and SE values associated with the disappearance of EPs in the EEGs in the VIMA-EP and VCRII-EP groups and the highest RE and SE values in the EEGs in the VIMA-nEP and VCRII-nEP groups (Table 1). The RE and SE values at these time points (stages 1–5) are presented on the graph (Figure 1 and Figure 2). 

The influence of induction on the levels of these parameters at individual stages in the study is presented in Table 1 and Table A1. There were no significant differences in baseline values in any of the groups. No significant differences were observed among individual groups for the parameters FeAA, FiAA, MAC, HR, SAP, DAP, MAP, and fEMG at the different stages of the study. Significant differences in SE and RE values in groups were noted in the third and fifth stages. At the third stage of the study, the highest values of SE and RE were observed in patients with no EPs in their EEGs, wherein the VCRII technique was utilised (VCRII-nEP group), while the differences at the fifth stage were observed in patients undergoing induction using the VIMA technique with EPs present.

There was a significant decline in the SAP, MAP, fEMG, SE, and RE values in patients induced using the VCRII technique with no EPs (VCRII-nEP group) in the final stages of the study (stages 3, 4, 5). The lower fEMG values at the fourth and fifth stages were noted in patients induced using the VIMA technique with EPs in their EEGs, a significant increase in the value at the final stage in the case of SE and RE was observed (Table 2, Table A2). 

**Figure 3:**
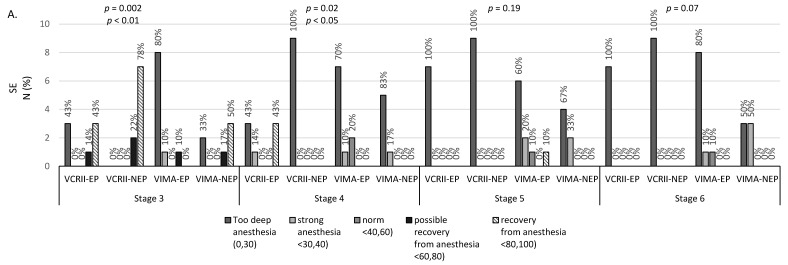

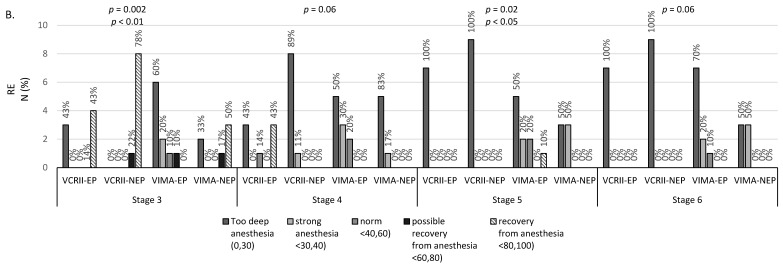
Number of erroneous (**A**) SE, (**B**) RE values at different stages of general anaesthesia. SE—state entropy, RE—response entropy. Results presented as numbers (percentages) for discrete variables.

We observed normative values of SE and RE (40–60) in less than 10% of anaesthetised patients regardless of the analysed stage of VIGA. A significantly higher percentage of patients who possibly recovered from anaesthesia (based on the SE and RE values) among the patients induced using the VCRII technique was observed at the third stage, while most of those in deep anaesthesia at this stage belonged to the VIMA group. For all patients induced with the VCRII technique, we noted deep anaesthesia (based on the parameter RE) at the fifth stage of the study (Figure 3). 

Among patients induced using the VCRII technique and showing EPs (VCRII-EP group), significantly higher values of SE and RE were associated with higher values of HR. Significant negative correlations between SE and SAP, DAP and MAP were observed for the patients who were induced with the VCRII technique and showed no EPs (VCRII-nEP). In the case of RE, correlation parameters were related to DAP and MAP. Negative correlation of RE with fiAA values was noted in patents induced with VCRII technique and showing the development of EPs (VCRII-EP group) (Table 3). 

During the presence of EPs, PS and PSR that may falsely indicate intraoperative awareness were present regardless of the anaesthetic regimen used, whereas PEDs appeared mainly during SE values that may falsely indicate deep anaesthesia (Table 4). 

**Figure 4:**
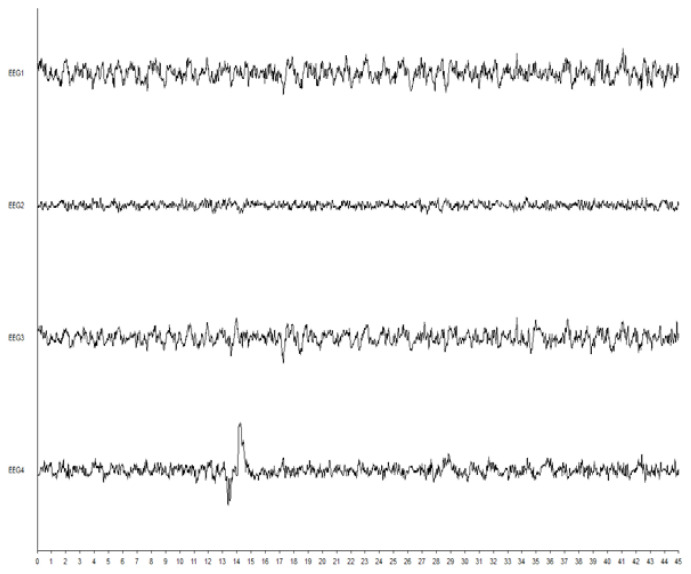
Epileptiform activity during increase in RE and SE values at fifth stage of VIGA with sevoflurane using VIMA technique.

Interestingly, in one male patient aged 41 years (weight, 61 kg; height, 164 cm) induced with sevoflurane using the VIMA technique, before the appearance of EPs (stage 3), the values of SE and RE were 60 and 79, respectively (FeAA: 5.22%, FiAA: 5.83%, MAC: 2.54, SAP: 105 mmHg, DAP: 60 mmHg, MAP: 78 mmHg, HR: 80 beats/min, and fEMG: 0.6). When PS appeared in his EEG recordings, during the first 10 s, the SE and RE values decreased to 45 and 59, respectively (FeAA: 4.19%, FiAA: 5.80%, MAC: 2.04, SAP: 105 mmHg, DAP: 60 mmHg, MAP: 78 mmHg, HR: 81 beats/min, and fEMG: 0.4) and subsequently increased to 80 and 94, respectively, 90 s later (FeAA: 5.22%, FiAA: 5.83%, MAC: 2.54, SAP: 105 mmHg, DAP: 60 mmHg, MAP: 78 mmHg, HR: 80 beats/min, fEMG: 0.3), indicating false recovery from anaesthesia, whereas the depth of anaesthesia measured by clinical parameters did not change during the transition from stages 3 to 4 and 5. Epileptiform activity during the abovementioned increase in RE and SE is presented in Figure 4. It persisted in this patient’s EEG recordings for a further 540 s until NMBD administration, so the later EEG patterns were not further analysed. 

**Figure 5:**
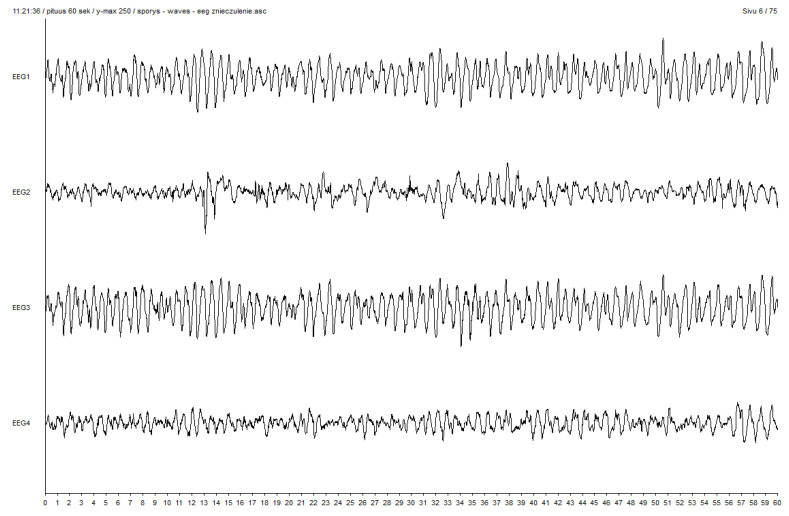
Epileptiform activity during the lowest values of RE and SE at fifth stage of VIGA with sevoflurane using VIMA technique.

Surprisingly, in another male patient aged 23 years, 140 s after induction was started using VIGA with sevoflurane (stage 1) and 50 s after LOC (stage 2), we observed convulsions following tonic-clonic generalized seizures. Further EEG pattern analysis revealed the development of spiky activity. In this case, evident epileptiform activity in the patient’s EEG recordings accompanied by clinically manifested tonic-clonic seizures that did not result in a rapid increase of RE and SE values, because the observed values of RE and SE were 7–18 and 7–15, respectively, indicating toxic concentrations of sevoflurane in the ventilation gas, whereas at the time of the lowest RE and SE values (RE: 7, SE: 7), the FeAA, FiAA, and MAC values were 4.52, 7.62, and 2.20, respectively (Figure 5).

## 4. Discussion

In our study, induction of VIGA with sevoflurane resulted in the presence of EPs in patients’ EEGs at a similar rate to previous studies concerning the presence of EPs during GA with sevoflurane [1,2,3,5,6,7,26,27]. We observed significantly higher HR values in patients induced with the VCRII technique when the EPs appeared; therefore, their appearance resulted in haemodynamic instability, since patients who were induced with the VCRII technique but did not show EPs showed lower haemodynamic parameters at the same stages (Table 3). Our study findings are consistent with those reported by Yli-Hankala A et al. [1] and Vakkuri A et al. [2], who also observed haemodynamic instability during the presence of EPs in patients’ EEG recordings. 

To our knowledge, the problems associated with aberrant values of EEG-based indices in the presence of EPs have been quite well recognized in terms of BIS [6,26,27], but there are only a few studies concerning the influence of EPs on entropy values during GA with sevoflurane. Some recent studies have made elaborate attempts to distinguish between normal and epileptic EEG signals by more advanced nonlinear entropy methods in comparison with the conventional entropy methods [40,41,42,43,44]. Entropy is being more successfully utilised in detection of epileptiform activity, showing acceptable performance for all three classification problems (interictal EEG from normal, ictal EEG from normal, and ictal EEG from interictal) in epileptic patients. 

Visual inspection of the EEG signals for detection of normal, interictal, and ictal activities is a challenging task due to the huge volumes of EEG segments that have to be studied offline or the speed of EEG pattern changes when monitoring online during GA. Therefore, attempts have been made to utilise non-linear methods to study EEG signals for automatic monitoring of epileptic activities. The ultimate goal of clinical anaesthesiologists is to develop a computerized technique that can classify all the three classes of EEG segments (normal, interictal and ictal), and the need for such software directs current research to investigate the possibility of application of automatic seizure monitoring in the near future, which could provide a real-time EEG-based brain monitoring system for epileptic seizure prediction [44].

The exemplary studies in this field investigating either the utility of observance of distribution entropy values [40] sample and multiscale entropy values [45,46] during transition between non-epileptiform and epileptiform stages reveal their rapid changes, what was consistent with the findings from our study. 

In our study on the fifth stage after induction using the VIMA technique, where EPs were present, RE and SE values were significantly higher than those in patients who showed no EPs in their EEGs at the same stage, similar to the findings obtained by Li P et al. [40]. 

At the third stage of the study analysis (before the appearance of EPs in patients’ EEGs), higher values of SE and RE were observed in the group of patients with EPs in their EEGs after induction using the VCRII technique (VCRII-nEP group), in comparison to stages 4 (first 10 s of the presence of EPs) and 5, when RE and SE values where the highest in the presence of EPs, similar to findings of Lu WY et al. [45]. 

Rapid decrease in RE and SE in the VCRII-EP group during the transition from stage 3 to 4 in the current study analysis, and contrary to our observation in the VIMA-EP group, where during the transition from stage 3 to 4, such reductions were not present were also observed by Weng WC et al. [46]. 

Globally, at the third stage of VIGA with the potential presence of EPs, 13 patients presented with SE values indicating recovery from GA, 5 patients showed SE values indicating potential recovery from GA with recall, whereas no patient showed values indicating a surgical level of anaesthesia, and 13 patients showed values indicating almost toxic concentrations of sevoflurane in the ventilation gas. However, no differences in FiAA, FeAA, and MAC were observed at this time point. At the fourth stage in 3 patients from the VCRII group, after appearance of EPs, both RE and SE values indicated potential recovery from GA, but in patients with no EPs in their EEGs, such values were not observed. Therefore, we conclude that the appearance of EPs may elevate RE and SE values falsely, falsely indicating insufficient concentration of sevoflurane in ventilation gas. Interestingly, in 24 patients, the SE values represented almost toxic concentrations of sevoflurane that almost doubled during the transition from stage 3 to 4 with no corresponding changes in FiAA, FeAA and MAC. At the fifth stage, in one patient from the VIMA group who showed EPs, both RE and SE values elevated to a level corresponding to recovery from GA. For almost 30 patients, these values stayed < 40, indicating too deep anaesthesia, with no statistically significant changes in FiAA, FeAA and MAC during the transition from stage 4 to 5; this was identical to our observations for a patient presenting with generalised tonic-clonic seizures whose RE and SE values were <20 during spiky activity in his EEG recordings despite moderate FiAA, FeAA, and MAC at this time. 

Monitors showing EEG-based indices, which evaluate the hypnotic component of anaesthesia by analysing patients’ EEGs, proved helpful in decreasing the number of incidences of intraoperative awareness with recall [47]. On the other hand, it must be emphasized that the main limitation of the study analysis is that we were not sure if RE and SE truly display reliable values online not only due to possible influence of the presence of EPs, so our analysis becomes speculative to some extent. Narcotrend and BIS were proven to require different time delays lasting up to even 60 s before calculation of an index representing the anaesthetic level until the correct index is displayed [10,38]. Kreuzer M et al. [48] in their study determined the time delays for SE and reported that the time delays were not constant and ranged from 18 to 152 s, and were also different for increasing and decreasing values and depended on the starting and target index values. They concluded that time delays in SE index calculations may limit its ability to prevent intraoperative awareness with recall. 

We observed that a significantly higher percentage of patients falsely recovered from anaesthesia (based on the SE and RE values) among the patients induced using VCRII technique at the third stage (before EPs appeared), while among those in deep anaesthesia, most patients were induced using the VIMA technique. We suppose that abovementioned difference might have been caused by a shift in transformation of the EEG signal detected by a frontal sensor into RE and SE value in patients who underwent VIGA with the VCRII technique, which is very fast. Conversely, such correlations were not observed in the VIMA group since induction of VIGA in these patients was relatively slow, so the shift in transformation of EEG signal into RE and SE value might have been of no clinical relevance.

In our study, EPs appeared on average after 209 ± 195 s after the onset of VIGA in patients anesthetized using VCRII. In three patients from the VCRII group, RE and SE values reflected potential false recovery from anaesthesia during the presence of EPs in their EEGs, whereas in patients anesthetized using the same technique who showed no EPs in their EEG at the fourth stage (VCRII-nEP group), all patients had RE values < 40 and SE values < 30, possibly falsely indicating toxic concentrations of sevoflurane in the ventilation gas. At the fifth stage, the SE values decreased below 40, mainly falsely representing a toxic level of anaesthesia (SE < 30 in 26 patients) in the VCRII-EP group despite the continuous presence of PS-type EPs. Surprisingly, in one patient anesthetized using the VIMA technique, at fifth stage of VIGA, SE and RE increased to >80 during the presence of PS-type EPs, possibly falsely indicating recovery from anaesthesia despite the steadily high concentration of volatile anaesthetic in ventilation gas at stages 3, 4 and 5. In this case, a potential shift in transformation of the EEG signal into a digital form is rather not likely to explain the sudden increase in RE and SE values, as at the fourth stage their indicated surgical level of anaesthesia (SE: 45, RE: 59) and concentration of sevoflurane in ventilation gas was not higher than that in the previous stages. 

Since the RE and SE values in a huge majority of patients at stage 5 reflect toxic concentrations of sevoflurane, regardless of technique used and despite the absence of changes in FeAA, FiAA, and MAC of volatile anaesthetics, further studies are required to investigate if RE and SE values may not falsely show too deep anaesthesia than it really is. Similar observations were obtained Laitio RM et al. [49] who observed lower state entropy values than BIS values during xenon anaesthesia, which is somehow similar to propofol anaesthesia, and reported the presence of high-amplitude delta activity and concluded that the power in the EEG delta band could have had a greater influence on SE values than on BIS values. It is of course questionable which of these two EEG-based indices show reliable values–higher BIS index or lower SE index–as such analyses are speculative. 

If SE values unreliably show too deep a level of anaesthesia as a result of the contribution of high-amplitude delta activity, it may possibly be hazardous for the patient because the anaesthesiologist could decrease the concentration of sevoflurane in ventilation gas and unintentionally awake the patient, if depth of anaesthesia is blindly guided based on observance of RE and SE values only. On the other hand, the time needed for transformation of EEG signal derived from frontal sensor might be the explanation for the fact that the concentration of end-expiratory sevoflurane increases faster during VIGA than the values of RE and SE decrease, and there may be a time delay in the reflection of the actual depth of anaesthesia in a huge majority of cases regardless of the appearance of EPs in patients EEGs, especially when the VCRII technique is utilised. Therefore, we suggest that further studies are needed to investigate if RE and SE reliably reflect the actual depth of anaesthesia during rapid changes of sevoflurane concentration in the end-expiratory gas by comparing values of different EEG-based indices that are popularly used to monitor depth of anaesthesia, and comparing them with EEG patterns, especially during the presence of PS-type EPs in patients’ EEGs. On the margin, in all patients who had EPs in their EEGs, we observed patterns of the PED type, but they tended to appear after NMBD administration, so we did not perform analysis afterwards. NMBD are known to influence values displayed by depth of anaesthesia monitors, which is well-recognised in the case of BIS scores [39,50,51,52]. Therefore, such analyses could be of no value as long as there is no study proving that there is no effect of administration of cis-atracurium on the SE and RE values, which might be a limitation of such analysis. Additionally, fEMG activity may possible also influence RE and SE values, since an increase in fEMG activity is known to lead to erroneous increase in BIS values [53,54] as well as RE and SE values [55]. In our study, fEMG values were low and showed a declining tendency during the transition between stage 3 to 4 and 4 to 5, regardless of group allocation, probably due to the fact that we did not hyperventilate patients in our study. 

In the end, since a lag in time is probably needed for transformation of the EEG signal recorded from a frontal sensor into a digital form displayed on the monitor, this factor could constitute the main limitation of the present study’s assessments, especially concerning VCRII group. Therefore, from a more practical point of view, we recommend on-line observation of variations in RE and SE values as well as FeAA, FiAA, and MAC of volatile anaesthetics, especially during the VCRII technique, because their verification against raw online EEG patterns in everyday practise is not available. Relying only on RE and SE values to guide the depth of anaesthesia may possibly lead to making a wrong decision resulting with either too deep anaesthesia or intraoperative awareness with recall. 

## 5. Conclusions

During different stages of VIGA with sevoflurane, RE and SE values may vary, showing a wide variety of values from >80, which may falsely represent recovery from anaesthesia during presence of EPs in patients’ EEGs, to <30, which may falsely indicate a toxic concentration of sevoflurane, regardless of the steady concentration of sevoflurane in the ventilation gas. The values of SE and RE during the presence of EPs in patient’s EEGs indicating false recovery from anaesthesia were associated with PS, whereas the presence of PEDs did not result in the presence of aberrant RE and SE values. RE and SE values during the presence of EPs are more likely to indicate toxic depth of anaesthesia rather than false recovery from anaesthesia with no change in FiAA, FeAA of sevoflurane, and MAC values between stages. Abovementioned aberrant RE and SE values could mislead anaesthesiologists into changing sevoflurane levels in the ventilation gas and either possibly lead to unintentional either recovery from anaesthesia or administering toxic concentration of sevoflurane, if depth of VIGA with sevoflurane is blindly guided based on observance of RE and SE values only. Further studies are required to investigate the behaviour of EEG-based indices during rapid changes in sevoflurane concentration at different stages of general anaesthesia and the influence of EPs on the transformation of EEG signals into a digital form. Therefore, during VIGA with sevoflurane, we recommend monitoring raw EEG in scientific studies to correlate it with the values of RE and SE and the end-tidal concentration of sevoflurane in everyday clinical practice, when monitoring raw EEG is not available.

## Figures and Tables

**Table 1:** Comparison of values of monitored patients’ parameters at the same stage between studied groups (stage 1—before onset of VIGA, stage 2—after LOC, stage 3—before presence of first epileptiform pattern, stage 4—during presence of epileptiform patterns, stage 5—during the highest values of RE and RE during presence of epileptiform patterns in patients from group VIMA and VCRII, Stage 6—during the highest values of RE and RE after disappearance of epileptiform patterns in patients from groups VIMA and VCRII).

Induction		VIMA-EP *N* = 10	VCRII-EP *N* = 7	VIMA-nEP *N* = 6	VCRII-nEP *N* = 9	*p*-Value ^a^
Level	Parameters					
STAGE 1	SE START	88.8 ± 1.6 89 (2)	88.1 ± 1.8 88 (3)	89.3 ± 0.8 89.5 (1)	88.3 ± 1 89 (1)	*p* = 0.41 NS
	RE START	97.7 ± 1.3 98 (1)	98.3 ± 0.8 98 (1)	98.5 ± 0.8 99 (1)	98.4 ± 0.7 99 (1)	*p* = 0.29 NS
STAGE 2	SE DURING LOC	85 ± 4.7 87 (3)	85.9 ± 4.9 87 (2)	85.8 ± 2 86 (1)	88.2 ± 1.9 89 (2)	*p* = 0.07 NS
	RE DURING LOC	95.5 ± 3.9 96.5 (2)	95.9 ± 4.1 98 (3)	96.3 ± 2.9 97.5 (3)	96.8 ± 2.2 97 (2)	*p* = 0.95 NS
STAGE 3	SE BEFORE EP	26.1 ± 14.5 25 (14)	53.4 ± 39.1 75 (77)	61 ± 29.8 72 (56)	82.1 ± 9.6 87 (9)	*p* = 0.004 *p* < 0.01 ^A^
	RE BEFORE EP	30 ± 20 27 (16)	59.1 ± 44 87 (85)	67.2 ± 33.3 80 (67)	90 ± 10 91 (7)	*p* = 0.009 *p* < 0.01 ^A^
STAGE 4	SE ONSET OF EP	24 ± 11.7 21.5 (16)	46.9 ± 38.4 33 (75)	22.5 ± 6 20 (8)	15.1 ± 5.8 13 (2)	*p* = 0.11 NS
	RE ONSET OF EP	29 ± 16 26.5 (22)	53.4 ± 41.4 44 (84)	24.5 ± 5 23 (6)	17.7 ± 7.8 16 (6)	*p* = 0.16 NS
STAGE 5	SE DURING EP	30 ± 20.1 21.5 (17)	10.9 ± 3.3 11 (5)	27 ± 6.7 25.5 (13)	18.6 ± 5.6 20 (4)	*p* = 0.001 *p* < 0.01 ^B^^,D^
	RE DURING EP	35.9 ± 24.3 27.5 (27)	12.3 ± 3.5 12 (5)	29 ± 7 30 (13)	19.6 ± 6.4 21 (6)	*p* = 0.001 *p* < 0.01 ^B^^,D^
STAGE 6	SE AFTER EP	24.1 ± 11.2 23 (8)	14.9 ± 2.6 15 (3)	28.2 ± 8.1 29.5 (14)	22.9 ± 3.7 22 (4)	*p* = 0.02 *p* < 0.05 ^D,E^
	RE AFTER EP	25.5 ± 12 23.5 (14)	14.9 ± 2.6 15 (3)	29.5 ± 7.1 30 (10)	24.2 ± 3.6 25 (4)	*p* = 0.005 *p* < 0.01 ^D,E^

FeAA—fraction of expired anaesthetic agent (sevoflurane), FiAA—fraction of inpired anaesthetic agent (sevoflurane), MAC—mean alveolar concentration of anaesthetic agent (sevoflurane), HR—heart rate, SAP—systolic arterial pressure, MAP—mean arterial pressure, DAP—diastolic arterial pressure, fEMG—facial electromyography, SE—state entropy, RE—response entropy. Results presented as means ± standard deviations and medians (interquartile ranges). ^a^ One-way analysis of variance (ANOVA). ^A^ Significantly higher in Group VCRII-nEP than in Group VIMA-EP (*p* < 0.01); ^B^ significantly higher in Group VIMA-EP than in Group VCRII-EP (*p* < 0.01); ^D^ significantly higher in Group VIMA-nEP than in Group VCRII-EP (*p* < 0.01); ^E^ significantly higher in Group VCRII-nEP than in Group VCRII-EP (*p* < 0.05).

**Table 2:** Comparison of values of monitored patients’ parameters between different stages of volatile induction of general anaesthesia in studied groups of patients.

Parameters	VIMA-EP				VCRII-EP				VIMA-nEP				VCRII-nEP				*p*-Value ^b^
	Stage			*p*-Value ^a^	Stage			*p*-Value ^a^	Stage			*p*-Value ^a^	Stage			*p*-Value ^a^	
	3	4	5		3	4	5		3	4	5		3	4	5		
SE	26.1 ± 14.5 25 (14)	24 ± 11.7 21.5 (16)	30 ± 20.1 21.5 (17)	*p* = 0.03 *p* < 0.05	53.4 ± 39.1 75 (77)	46.9 ± 38.4 33 (75)	10.9 ± 3.3 11 (5)	*p* = 0.20 NS	61 ± 29.8 72 (56)	22.5 ± 6 20 (8)	27 ± 6.7 25.5 (13)	*p* = 0.15 NS	82.1 ± 9.6 87 (9)	15.1 ± 5.8 13 (2)	18.6 ± 5.6 20 (4)	*p* = 0.0006 *p* < 0.01	*p* = 0.002 *p* < 0.01
RE	30 ± 20 27 (16)	29 ± 16 26.5 (22)	35.9 ± 24.3 27.5 (27)	*p* = 0.02 *p* < 0.05	59.1 ± 44 87 (85)	53.4 ± 41.4 44 (84)	12.3 ± 3.5 12 (5)	*p* = 0.34 NS	67.2 ± 33.3 80 (67)	24.5 ± 5 23 (6)	29 ± 7 30 (13)	*p* = 0.15 NS	90 ± 10 91 (7)	17.7 ± 7.8 16 (6)	19.6 ± 6.4 21 (6)	*p* = 0.0001 *p* < 0.001	*p* = 0.001 *p* < 0.01

FeAA—fraction of expired anaesthetic agent (sevoflurane), FiAA—fraction of inspired anaesthetic agent (sevoflurane), MAC—mean alveolar concentration of anaesthetic agent (sevoflurane), HR—heart rate, SAP—systolic arterial pressure, MAP—mean arterial pressure, DAP—diastolic arterial pressure, fEMG—facial electromyography, SE—state entropy, RE—response entropy. Results presented as means ± standard deviations X ± SD and medians (interquartile ranges) M (IQR). ^a^ One-way analysis of variance (ANOVA) for repeated measures. ^b^ Two-way analysis of variance (ANOVA) for repeated measures: Stage × Group

**Table 3:** The strength of the relationship between selected parameters. R’ Spearman’s correlation coefficient.

Group	Parameter	SE R’-Spearman *p*-Value ^a^				RE R’-Spearman *p*-Value ^a^			
		Stage				Stage			
		3	4	5	6	3	4	5	6
VCRII-EP	FeAA	−0.16 *p* = 0.73	−0.46 *p* = 0.29	−0.27 *p* = 0.55	−0.54 *p* = 0.21	−0.11 *p* = 0.82	−0.43 *p* = 0.34	−0.2 *p* = 0.67	−0.54 *p* = 0.21
	FiAA	−0.23 *p* = 0.61	0.68 *p* = 0.09	**−0.85** ***p* = 0.01**	−0.09 *p* = 0.85	−0.32 *p* = 0.48	0.54 *p* = 0.22	**−0.87** ***p* = 0.01**	−0.09 *p* = 0.85
	MAC	−0.16 *p* = 0.73	−0.46 *p* = 0.29	−0.27 *p* = 0.55	−0.54 *p* = 0.21	−0.11 *p* = 0.82	−0.43 *p* = 0.34	−0.2 *p* = 0.67	−0.54 *p* = 0.21
	HR	0.56 *p* = 0.19	**0.93** ***p* = 0.002**	−0.75 *p* = 0.05	−0.68 *p* = 0.09	0.54 *p* = 0.22	0.89 *p* = 0.01	−0.65 *p* = 0.11	−0.68 *p* = 0.09
	SAP	0.61 *p* = 0.14	0.71 *p* = 0.07	−0.29 *p* = 0.53	0.11 *p* = 0.82	0.54 *p* = 0.22	0.61 *p* = 0.15	−0.24 *p* = 0.61	0.11 *p* = 0.82
	DAP	0.09 *p* = 0.85	0.61 *p* = 0.15	0.07 *p* = 0.88	0.2 *p* = 0.67	0 *p* = 1	0.5 *p* = 0.25	0.05 *p* = 0.91	0.2 *p* = 0.67
	MAP	0.25 *p* = 0.59	0.68 *p* = 0.09	−0.15 *p* = 0.76	0.11 *p* = 0.82	0.14 *p* = 0.76	0.57 *p* = 0.18	−0.15 *p* = 0.76	0.11 *p* = 0.82
	fEMG	−0.18 *p* = 0.7	−0.23 *p* = 0.61	0.15 *p* = 0.76	−0.08 *p* = 0.86	−0.14 *p* = 0.76	−0.14 *p* = 0.76	0.2 *p* = 0.67	−0.08 *p* = 0.86
VCRII-nEP	FeAA	0.25 *p* = 0.51	0.3 *p* = 0.43	0.46 *p* = 0.21	−0.58 *p* = 0.1	0.34 *p* = 0.37	0.46 *p* = 0.21	0.3 *p* = 0.44	−0.34 *p* = 0.36
	FiAA	−0.34 *p* = 0.38	−0.54 *p* = 0.14	−0.58 *p* = 0.1	−0.37 *p* = 0.33	−0.55 *p* = 0.13	−0.25 *p* = 0.51	−0.44 *p* = 0.23	−0.34 *p* = 0.36
	MAC	0.25 *p* = 0.51	0.33 *p* = 0.39	0.46 *p* = 0.21	−0.59 *p* = 0.09	0.34 *p* = 0.37	0.46 *p* = 0.22	0.3 *p* = 0.44	−0.36 *p* = 0.34
	HR	**0.71** ***p* = 0.03**	−0.19 *p* = 0.62	0.43 *p* = 0.25	0.09 *p* = 0.82	0.52 *p* = 0.15	−0.05 *p* = 0.91	0.53 *p* = 0.14	0 *p* = 0.99
	SAP	−0.1 *p* = 0.8	**−0.69** ***p* = 0.04**	−0.08 *p* = 0.83	−0.18 *p* = 0.64	−0.38 *p* = 0.31	−0.57 *p* = 0.11	−0.12 *p* = 0.76	0.01 *p* = 0.98
	DAP	−0.08 *p* = 0.85	**−0.8** ***p* = 0.01**	0.22 *p* = 0.57	0.05 *p* = 0.9	−0.29 *p* = 0.45	−0.69 *p* = 0.04	0.12 *p* = 0.76	0.17 *p* = 0.67
	MAP	−0.13 *p* = 0.73	**−0.8** ***p* = 0.01**	−0.11 *p* = 0.78	−0.09 *p* = 0.81	−0.39 *p* = 0.3	−0.69 *p* = 0.04	−0.18 *p* = 0.65	0.12 *p* = 0.76
	fEMG	0.57 *p* = 0.11	0.6 *p* = 0.09	0.33 *p* = 0.39	0.36 *p* = 0.34	0.64 *p* = 0.06	0.6 *p* = 0.09	0.55 *p* = 0.13	0.36 *p* = 0.34
VIMA-EP	FeAA	−0.04 *p* = 0.91	−0.12 *p* = 0.75	−0.62 *p* = 0.06	−0.3 *p* = 0.39	−0.22 *p* = 0.54	−0.11 *p* = 0.76	−0.47 *p* = 0.17	−0.37 *p* = 0.29
	FiAA	−0.14 *p* = 0.7	−0.38 *p* = 0.28	−0.57 *p* = 0.08	−0.33 *p* = 0.35	−0.04 *p* = 0.92	−0.44 *p* = 0.21	−0.43 *p* = 0.22	−0.36 *p* = 0.31
	MAC	−0.04 *p* = 0.91	0.25 *p* = 0.49	−0.39 *p* = 0.27	−0.3 *p* = 0.39	−0.22 *p* = 0.54	0.24 *p* = 0.5	−0.28 *p* = 0.44	−0.37 *p* = 0.29
	HR	−0.19 *p* = 0.59	0.03 *p* = 0.93	−0.1 *p* = 0.79	−0.06 *p* = 0.87	−0.34 *p* = 0.34	0.17 *p* = 0.64	0.02 *p* = 0.96	0.01 *p* = 0.99
	SAP	−0.07 *p* = 0.85	−0.37 *p* = 0.29	−0.27 *p* = 0.45	−0.13 *p* = 0.73	0.05 *p* = 0.89	−0.52 *p* = 0.12	−0.5 *p* = 0.14	−0.21 *p* = 0.56
	DAP	−0.53 *p* = 0.12	−0.16 *p* = 0.65	−0.1 *p* = 0.77	−0.24 *p* = 0.5	−0.35 *p* = 0.33	−0.3 *p* = 0.39	−0.37 *p* = 0.29	−0.32 *p* = 0.37
	MAP	−0.09 *p* = 0.8	−0.2 *p* = 0.58	−0.1 *p* = 0.77	−0.2 *p* = 0.58	0.09 *p* = 0.82	−0.35 *p* = 0.32	−0.32 *p* = 0.36	−0.27 *p* = 0.45
	fEMG	0.06 *p* = 0.87	−0.03 *p* = 0.94	−0.11 *p* = 0.76	−0.15 *p* = 0.69	0.21 *p* = 0.56	0.07 *p* = 0.84	0.06 *p* = 0.87	−0.19 *p* = 0.59
VIMA-nEP	FeAA	0.37 *p* = 0.47	−0.03 *p* = 0.96	−0.2 *p* = 0.7	−0.67 *p* = 0.15	0.46 *p* = 0.35	0.06 *p* = 0.91	−0.03 *p* = 0.96	−0.71 *p* = 0.11
	FiAA	−0.6 *p* = 0.21	0.46 *p* = 0.35	−0.61 *p* = 0.2	−0.38 *p* = 0.46	−0.49 *p* = 0.32	0.17 *p* = 0.74	−0.49 *p* = 0.32	−0.49 *p* = 0.33
	MAC	0.37 *p* = 0.47	0.26 *p* = 0.62	−0.2 *p* = 0.7	−0.67 *p* = 0.15	0.46 *p* = 0.35	−0.06 *p* = 0.91	−0.03 *p* = 0.96	−0.71 *p* = 0.11
	HR	0.26 *p* = 0.62	0.29 *p* = 0.58	0.43 *p* = 0.39	−0.26 *p* = 0.61	0.14 *p* = 0.78	−0.23 *p* = 0.66	0.67 *p* = 0.15	−0.29 *p* = 0.58
	SAP	−0.2 *p* = 0.7	−0.32 *p* = 0.54	−0.17 *p* = 0.74	−0.81 *p* = 0.05	−0.12 *p* = 0.83	−0.17 *p* = 0.74	−0.23 *p* = 0.66	−0.77 *p* = 0.07
	DAP	−0.37 *p* = 0.47	−0.32 *p* = 0.54	−0.03 *p* = 0.96	**−0.9** ***p* = 0.01**	−0.35 *p* = 0.5	−0.75 *p* = 0.08	0.14 *p* = 0.78	**−0.94** ***p* = 0**
	MAP	−0.2 *p* = 0.7	−0.49 *p* = 0.32	0.12 *p* = 0.83	**−0.9** ***p* = 0.01**	−0.12 *p* = 0.83	−0.41 *p* = 0.42	0.23 *p* = 0.66	**−0.94** ***p* = 0**
	fEMG	0.35 *p* = 0.5	−0.28 *p* = 0.59	0.02 *p* = 0.98	−0.05 *p* = 0.92	0.35 *p* = 0.49	−0.92 *p* = 0.01	0.14 *p* = 0.79	−0.17 *p* = 0.75

^a^ One-way analysis of variance (ANOVA).

**Table 4:** Number of erroneous SE values including number of epileptiform patterns at different stages in groups with symptoms of epilepsy.

Parameter	Stage 4					Stage 5					Stage 6				
SE N/%	Polispykes (PS) and Rhytmic Polispykes (RPS)				Periodic Epileptiform Discharges (PED)	Polyspikes (PS) and Rhytmic Polispykes (RPS)				Periodic Epileptiform Discharges (PED)	Polyspikes (PS) and Rhytmic Polispykes (RPS)				Periodic Epileptiform Discharges (PED)
	VCRII-EP	VIMA-EP	Total	*p*-Value ^a^	VIMA-EP	VCRII-EP	VIMA-EP	Total	*p*-Value ^a^	VIMA-EP	VCRII-EP	VIMA-EP	Total	*p*-Value ^a^	VIMA-EP
deep anesthesia	3	4	7	*p* = 0.23 NS	3	7	3	10	*p* = 0.15 NS	3	7	4	11	*p* = 0.17 NS	4
(0.30)	42.9%	80.0%	58.3%		60%	100.0%	60.0%	83.3%		60%	100%	80%	91.7%		80%
strong anesthesia	1	0	1		1	0	1	1		1	0	0	0		1
<30.40)	14.3%	0.0%	8.3%		20%	0.0%	20.0%	8.3%		20%	0%	0%	0%		20%
Norm	0	1	1		1	0	0	0		1	0	1	1		0
<40.60)	0.0%	20.0%	8.3%		20%	0.0%	0.0%	0.0%		20%	0%	20%	8.3%		0%
possible recovery from anesthesia	0	0	0		0	0	0	0		0	0	0	0		0
<60.80)	0.0%	0.0%	0.0%		0%	0.0%	0.0%	0.0%		0%	0%	0%	0%		0%
recovery from anesthesia	3	0	3		0	0	1	1		0	0	0	0		0
<80.100)	42.9%	0.0%	25.0%		0%	0.0%	20.0%	8.3%		0%	0%	0%	0%		0%

Results presented as numbers (percentages) for discrete variables. ^a^ the Fisher’s Exact Test.

## Appendix A

**Table A1:** Comparison of values of monitored patients’ parameters at the same stage between studied groups. (stage 1: before onset of VIGA, stage 2: after LOC, stage 3: before presence of first epileptiform pattern, stage 4: during presence of epileptiform patterns, stage 5: during the highest values of RE and RE during presence of epileptiform patterns in patients from group VIMA and VCRII, Stage 6: during the highest values of RE and RE after disappearance of epileptiform patterns in patients from group VIMA and VCRII).

Induction		VIMA-EP N = 10 X ± SD M (IQR)	VCRII-EP N = 7 X ± SD M (IQR)	VIMA-nEP N = 6 X ± SD M (IQR)	VCRII-nEP N = 9 X ± SD M (IQR)	*p*-Value ^a^
Level	Parameters					
STAGE 1	HR START (beats/min)	79.3±9.4 83 (13)	81.1 ± 17.3 73 (31)	66.2 ± 12.8 62 (19)	81.6 ± 17.5 82 (5)	*p* = 0.18 NS
	SAP START (mmHg)	151.1 ± 26 156.8 (34.3)	138.1 ± 11.7 134.3 (24.2)	143.2 ± 9.9 141 (8.8)	150.8 ± 36.1 143.4 (40.5)	*p* = 0.53 NS
	DAP START (mmHg)	89.2 ± 15.1 95.7 (19)	70.5 ± 10.2 72.1 (18.3)	82.4 ± 3.3 82.3 (3.2)	85.6 ± 18.1 85.5 (26)	*p* = 0.06 NS
	MAP START (mmHg)	113.7 ± 17.5 118.3 (18.2)	97.3 ± 9.1 98.3 (6.7)	104.9 ± 5.4 105.4 (3.8)	109.6 ± 24.1 104.6 (27.6)	*p* = 0.12 NS
	fEMG START	7 ± 4.7 6 (6.4)	13.5 ± 11.6 11.2 (20.7)	7.3 ± 6.6 6.3 (7.5)	7.5 ± 4.8 5.8 (4.4)	*p* = 0.79 NS
STAGE 2	FeAA DURING LOC	4.4 ± 1.4 4.7 (1.9)	3.8 ± 1.1 3.8 (2.3)	4.3 ± 1.5 4.8 (2.7)	4.8 ± 1.5 4.5 (2.1)	*p* = 0.53 NS
	FiAA DURING LOC	6.2 ± 1.7 6.6 (1.3)	6.9 ± 1.1 6.8 (1.6)	6.4 ± 1 6.6 (1.8)	7.5 ± 0.4 7.6 (0.2)	*p* = 0.12 NS
	MAC DURING LOC	2.1 ± 0.7 2.3 (0.9)	1.8 ± 0.5 1.8 (1.1)	2.1 ± 0.7 2.3 (1.3)	2.3 ± 0.7 2.2 (1)	*p* = 0.54 NS
	HR DURING LOC (beats/min)	84.9 ± 21.4 78.5 (34)	88 ± 17.2 81 (28)	77.3 ± 24 72 (25)	92.6 ± 22 96 (14)	*p* = 0.59 NS
	SAP DURING LOC (mmHg)	158.7 ± 34 155.1 (32.7)	138.9 ± 11 137.5 (22.3)	150.4 ± 22.1 150.4 (23.1)	149.9 ± 37.5 156 (41.4)	*p* = 0.61 NS
	DAP DURING LOC (mmHg)	86 ± 16.6 88.5 (21.2)	72.3 ± 9.7 74.7 (8.4)	86.9 ± 3.8 88 (2.7)	83.4 ± 17.4 81.5 (26.3)	*p* = 0.12 NS
	MAP DURING LOC (mmHg)	113.4 ± 19.1 114.8 (17)	95.6 ± 17.3 100.1 (9.9)	112 ± 10.9 112.6 (9.9)	107.6 ± 21.5 101.4 (27.4)	*p* = 0.20 NS
	fEMG DURING LOC	1.8 ± 1.5 1.2 (1.4)	15.4 ± 36.6 1.4 (2.4)	1.6 ± 1.4 1 (1.4)	1.3 ± 1 1.3 (1.3)	*p* = 0.62 NS
STAGE 3	FeAA BEFORE EP (%)	5.1 ± 0.9 5.2 (1)	4.9 ± 1.4 4.3 (2.2)	5 ± 0.4 5 (0.3)	4.8 ± 0.9 5 (1.2)	*p* = 0.92 NS
	FiAA BEFORE EP (%)	6.6 ± 0.8 6.7 (1.4)	6.4 ± 0.9 5.8 (1.6)	6.5 ± 0.9 6.4 (1.2)	7.1 ± 0.8 7.3 (0.9)	*p* = 0.45 NS
	MAC BEFORE EP	2.5 ± 0.4 2.5 (0.5)	2.4 ± 0.7 2.1 (1.1)	2.4 ± 0.2 2.5 (0.1)	2.3 ± 0.4 2.4 (0.6)	*p* = 0.92 NS
	HR BEFORE EP (beats/min)	80.4 ± 17 82.5 (23)	91.1 ± 20.2 85 (33)	79.7 ± 19.8 77 (14)	87.9 ± 16.9 92 (19)	*p* = 0.55 NS
	SAP BEFORE EP (mmHg)	137.2 ± 20.5 143.3 (24.7)	138.9 ± 11 137.5 (22.3)	140.7 ± 26.9 142.5 (41.7)	147.3 ± 40 138.1 (41.2)	*p* = 0.98 NS
	DAP BEFORE EP (mmHg)	78.4 ± 16.8 78.4 (27)	72.8 ± 8.5 74.7 (8.4)	82.3 ± 11.7 87.3 (8.9)	81.2 ± 16.6 79.4 (23.8)	*p* = 0.47 NS
	MAP BEFORE EP (mmHg)	102.8 ± 16.8 104 (21.5)	101.2 ± 6.6 100.1 (8.2)	105.3 ± 17.4 108.5 (21)	105.6 ± 22.9 97.1 (24.2)	*p* = 0.95 NS
	fEMG BEFORE EP	1.2 ± 2.1 0.3 (1.2)	0.8 ± 0.6 0.8 (0.8)	0.8 ± 0.5 0.9 (0.3)	0.9 ± 1.2 0.4 (0.7)	*p* = 0.82 NS
STAGE 4	FeAA ONSET OF EP (%)	4.6 ± 0.7 4.7 (0.9)	4.5 ± 0.9 4.4 (1)	5.7 ± 1.2 5.2 (2.3)	5 ± 1 4.9 (1)	*p* = 0.14 NS
	FiAA ONSET OF EP (%)	6.1 ± 0.9 6.2 (1.5)	6.8 ± 0.9 6.7 (1.9)	6 ± 1.5 6.3 (2.2)	6.8 ± 0.5 6.9 (0.3)	*p* = 0.30 NS
	MAC ONSET OF EP	2.3 ± 0.3 2.4 (0.5)	2.2 ± 0.5 2.2 (0.5)	2.7 ± 0.7 2.5 (1.3)	2.4 ± 0.5 2.4 (0.5)	*p* = 0.43 NS
	HR ONSET OF EP (beats/min)	81.9 ± 23 79 (24)	101 ± 32.2 94 (59)	78.5 ± 15.6 79 (9)	79.2 ± 18.8 83 (22)	*p* = 0.23 NS
	SAP ONSET OF EP (mmHg)	135.9 ± 26.1 126.2 (33.2)	122.9 ± 25.7 129.6 (30.7)	129.5 ± 14.2 128.7 (19.1)	130.7 ± 27.8 119.9 (34.9)	*p* = 0.94 NS
	DAP ONSET OF EP (mmHg)	80.2 ± 16.4 81.1 (19.7)	63.1 ± 13.4 70.2 (25)	80.7 ± 11.7 85.4 (13.9)	79.7 ± 17.4 77.8 (12.7)	*p* = 0.11 NS
	MAP ONSET OF EP (mmHg)	101.1 ± 20.4 98.3 (20.7)	89.4 ± 18.4 98.3 (24.9)	97.1 ± 10.8 99.7 (5.6)	98.4 ± 19.7 92.3 (14.4)	*p* = 0.84 NS
	fEMG ONSET OF EP	0.2 ± 0.1 0.2 (0.2)	0.6 ± 0.4 0.8 (0.7)	0.4 ± 0.6 0.2 (0.2)	0.3 ± 0.6 0.1 (0)	*p* = 0.09 NS
STAGE 5	FeAA DURING EP (%)	4.4 ± 1.5 4.5 (1.8)	5.2 ± 1.5 4.7 (2.3)	5.6 ± 1.6 6.1 (2)	5.3 ± 1 4.9 (1)	*p* = 0.36 NS
	FiAA DURING EP (%)	5.7 ± 1.7 6.2 (1.8)	7 ± 0.8 7 (1.2)	6.1 ± 1.5 6.7 (1.5)	6.6 ± 0.6 6.6 (0.4)	*p* = 0.21 NS
	MAC DURING EP	2.3 ± 0.7 2.3 (0.5)	2.6 ± 0.7 2.3 (1.1)	2.7 ± 0.8 2.9 (1)	2.6 ± 0.5 2.4 (0.5)	*p* = 0.53 NS
	HR DURING EP (beats/min)	82.9 ± 20.1 77.5 (22)	87.9 ± 18.2 82 (34)	76.8 ± 12.7 81 (20)	77.4 ± 15.6 76 (9)	*p* = 0.59 NS
	SAP DURING EP (mmHg)	132.9 ± 22.9 128.1 (31.1)	132.1 ± 13.3 132.1 (13.3)	126.7 ± 12.5 126.9 (5.8)	129.1 ± 34.2 119.9 (52)	*p* = 0.89 NS
	DAP DURING EP (mmHg)	80.4 ± 17.3 84.3 (23.6)	68.1 ± 9.9 71.3 (16.4)	78.8 ± 11 79.7 (13.9)	75.3 ± 20.9 76.4 (13.8)	*p* = 0.47 NS
	MAP DURING EP (mmHg)	101 ± 18.2 101.9 (20.7)	96.2 ± 9.6 98.6 (4.8)	95 ± 9 97.4 (5.6)	95.4 ± 23.4 92.3 (21)	*p* = 0.71 NS
	fEMG DURING EP	0.3 ± 0.2 0.2 (0.2)	0.5 ± 0.4 0.4 (0.8)	0.3 ± 0.6 0.1 (0.1)	0.3 ± 0.3 0.1 (0.2)	*p* = 0.08 NS
STAGE 6	FeAA AFTER EP (%)	4.1 ± 1.3 4.1 (2.1)	3.8 ± 1.1 3.9 (1.4)	4.7 ± 1 4.4 (0.9)	4.1 ± 0.9 4 (1.1)	*p* = 0.57
	FiAA AFTER EP (%)	5 ± 1.6 5.5 (2.4)	5 ± 1.6 5.1 (3.6)	5.5 ± 1.1 5.3 (1.7)	4.6 ± 0.9 4.6 (1.3)	*p* = 0.70
	MAC AFTER EP	2 ± 0.6 2 (1)	1.9 ± 0.5 1.9 (0.7)	2.3 ± 0.5 2.1 (0.4)	2 ± 0.4 2 (0.5)	*p* = 0.58
	HR AFTER EP (beats/min)	82.5 ± 23.8 77.5 (28)	82.3 ± 20.8 71 (38)	67 ± 15.5 69 (33)	81.7 ± 17.1 83 (26)	*p* = 0.44
	SAP AFTER EP (mmHg)	139.8 ± 40.9 134.7 (37.1)	107.9 ± 19.4 108.6 (32.7)	104.1 ± 26.3 108.7 (47.8)	111.8 ± 20.4 107.6 (25.5)	*p* = 0.07
	DAP AFTER EP (mmHg)	81.6 ± 23.1 84.3 (35.9)	54.9 ± 10.8 51.1 (15.6)	64.9 ± 20.8 65.1 (29.5)	65.9 ± 13 61.4 (24.5)	*p* = 0.03 *p* < 0.05 ^C^
	MAP AFTER EP (mmHg)	104 ± 29.6 101.9 (43.1)	80.5 ± 14.4 75.2 (25.1)	80.3 ± 21.2 83.9 (32.6)	83.9 ± 13.8 80.5 (19.9)	*p* = 0.08
	fEMG AFTER EP	0.2 ± 0.1 0.2 (0.2)	0.2 ± 0.1 0.2 (0.2)	0.2 ± 0.2 0.1 (0.1)	0.1 ± 0.1 0.1 (0)	*p* = 0.37

Legend: FeAA—fraction of expired anaesthetic agent (sevoflurane), FiAA—fraction of inpired anaesthetic agent (sevoflurane), MAC—mean alveolar concentration of anaesthetic agent (sevoflurane), HR—heart rate, SAP—systolic arterial pressure, MAP-mean arterial pressure, DAP—diastolic arterial pressure, fEMG—facial electromyography, SE—state entropy, RE-response entropy. Results presented as means ± standard deviations and medians (interquartile ranges). ^a^ 1-way analysis of variance (ANOVA). ^C^ Significantly higher in Group VIMA-EP than in Group VCRII-EP (*p* < 0.05).

**Table A2:** Comparison of values of monitored patients’ parametres between different stages of volatile induction of general anaesthesia in studied groups of patients.

Parameters	VIMA-EP X ± SD M (IQR)				VCRII-EP X ± SD M (IQR)				VIMA-nEP X ± SD M (IQR)				VCRII-nEP X ± SD M (IQR)				*p*-Value ^b^
	Stage			*p*-Value ^a^	Stage			*p*-Value ^a^	Stage			*p*-Value ^a^	Stage			*p*-Value ^a^	
	3	4	5		3	4	5		3	4	5		3	4	5		
FeAA	5.1 ± 0.9 5.2 (1)	4.6 ± 0.7 4.7 (0.9)	4.4 ± 1.5 4.5 (1.8)	*p* = 0.31 NS	4.9 ± 1.4 4.3 (2.2)	4.5 ± 0.9 4.4 (1)	5.2 ± 1.5 4.7 (2.3)	*p* = 0.58 NS	5 ± 0.4 5 (0.3)	5.7 ± 1.2 5.2 (2.3)	5.6 ± 1.6 6.1 (2)	*p* = 0.49 NS	4.8 ± 0.9 5 (1.2)	5 ± 1 4.9 (1)	5.3 ± 1 4.9 (1)	*p* = 0.46 NS	*p* = 0.43 NS
FiAA (%)	6.6 ± 0.8 6.7 (1.4)	6.1 ± 0.9 6.2 (1.5)	5.7 ± 1.7 6.2 (1.8)	*p* = 0.06 NS	6.4 ± 0.9 5.8 (1.6)	6.8 ± 0.9 6.7 (1.9)	7 ± 0.8 7 (1.2)	*p* = 0.63 NS	6.5 ± 0.9 6.4 (1.2)	6 ± 1.5 6.3 (2.2)	6.1 ± 1.5 6.7 (1.5)	*p* = 0.67 NS	7.1 ± 0.8 7.3 (0.9)	6.8 ± 0.5 6.9 (0.3)	6.6 ± 0.6 6.6 (0.4)	*p* = 0.09 NS	*p* = 0.30 NS
MAC	2.5 ± 0.4 2.5 (0.5)	2.3 ± 0.3 2.4 (0.5)	2.3 ± 0.7 2.3 (0.5)	*p* = 0.56 NS	2.4 ± 0.7 2.1 (1.1)	2.2 ± 0.5 2.2 (0.5)	2.6 ± 0.7 2.3 (1.1)	*p* = 0.58 NS	2.4 ± 0.2 2.5 (0.1)	2.7 ± 0.7 2.5 (1.3)	2.7 ± 0.8 2.9 (1)	*p* = 0.58 NS	2.3 ± 0.4 2.4 (0.6)	2.4 ± 0.5 2.4 (0.5)	2.6 ± 0.5 2.4 (0.5)	*p* = 0.46 NS	*p* = 0.82 NS
HR (beats /min)	80.4 ± 17 82.5 (23)	81.9 ± 23 79 (24)	82.9 ± 20.1 77.5 (22)	*p* = 0.80 NS	91.1 ± 20.2 85 (33)	101 ± 32.2 94 (59)	87.9 ± 18.2 82 (34)	*p* = 0.39 NS	79.7 ± 19.8 77 (14)	78.5 ± 15.6 79 (9)	76.8 ± 12.7 81 (20)	*p* = 0.91 NS	87.9 ± 16.9 92 (19)	79.2 ± 18.8 83 (22)	77.4 ± 15.6 76 (9)	*p* = 0.07 NS	*p* = 0.43 NS
SAP (mmHg)	137.2 ± 20.5 143.3 (24.7)	135.9 ± 26.1 126.2 (33.2)	132.9 ± 22.9 128.1 (31.1)	*p* = 0.72 NS	138.9 ± 11 137.5 (22.3)	122.9 ± 25.7 129.6 (30.7)	132.1 ± 13.3 132.1 (13.3)	*p* = 0.17 NS	140.7 ± 26.9 142.5 (41.7)	129.5 ± 14.2 128.7 (19.1)	126.7 ± 12.5 126.9 (5.8)	*p* = 0.45 NS	147.3 ± 40 138.1 (41.2)	130.7 ± 27.8 119.9 (34.9)	129.1 ± 34.2 119.9 (52)	*p* = 0.006 *p* < 0.01	*p* = 0.40 NS
DAP (mmHg)	78.4 ± 16.8 78.4 (27)	80.2 ± 16.4 81.1 (19.7)	80.4 ± 17.3 84.3 (23.6)	*p* = 0.93 NS	72.8 ± 8.5 74.7 (8.4)	63.1 ± 13.4 70.2 (25)	68.1 ± 9.9 71.3 (16.4)	*p* = 0.14 NS	82.3 ± 11.7 87.3 (8.9)	80.7 ± 11.7 85.4 (13.9)	78.8 ± 11 79.7 (13.9)	*p* = 0.82 NS	81.2 ± 16.6 79.4 (23.8)	79.7 ± 17.4 77.8 (12.7)	75.3 ± 20.9 76.4 (13.8)	*p* = 0.18 NS	*p* = 0.32 NS
MAP (mmHg)	102.8 ± 16.8 104 (21.5)	101.1 ± 20.4 98.3 (20.7)	101 ± 18.2 101.9 (20.7)	*p* = 0.94 NS	101.2 ± 6.6 100.1 (8.2)	89.4 ± 18.4 98.3 (24.9)	96.2 ± 9.6 98.6 (4.8)	*p* = 0.10 NS	105.3 ± 17.4 108.5 (21)	97.1 ± 10.8 99.7 (5.6)	95 ± 9 97.4 (5.6)	*p* = 0.25 NS	105.6 ± 22.9 97.1 (24.2)	98.4 ± 19.7 92.3 (14.4)	95.4 ± 23.4 92.3 (21)	*p* = 0.01 *p* < 0.05	*p* = 0.15 NS
fEMG	1.2 ± 2.1 0.3 (1.2)	0.2 ± 0.1 0.2 (0.2)	0.3 ± 0.2 0.2 (0.2)	*p* = 0.02 *p* < 0.05	0.8 ± 0.6 0.8 (0.8)	0.6 ± 0.4 0.8 (0.7)	0.5 ± 0.4 0.4 (0.8)	*p* = 0.50 NS	0.8 ± 0.5 0.9 (0.3)	0.4 ± 0.6 0.2 (0.2)	0.3 ± 0.6 0.1 (0.1)	*p* = 0.14 NS	0.9 ± 1.2 0.4 (0.7)	0.3 ± 0.6 0.1 (0)	0.3 ± 0.3 0.1 (0.2)	*p* = 0.02 *p* < 0.05	*p* = 0.12 NS

Legend: FeAA—fraction of expired anaesthetic agent (sevoflurane), FiAA—fraction of inpired anaesthetic agent (sevoflurane), MAC—mean alveolar concentration of anaesthetic agent (sevoflurane), HR—heart rate, SAP—systolic arterial pressure, MAP—mean arterial pressure, DAP – diastolic arterial pressure, fEMG – facial electromyography, SE—state entropy, RE—response entropy. Results presented as means ± standard deviations and medians (interquartile ranges). ^a^. 1-way analysis of variance (ANOVA) for repeated measures ^b^. 2-way analysis of variance (ANOVA) for repeated measures: Stage*Group.
